# Root-Associated Antagonistic Pseudomonas spp. Contribute to Soil Suppressiveness against Banana Fusarium Wilt Disease of Banana

**DOI:** 10.1128/spectrum.03525-22

**Published:** 2023-02-14

**Authors:** Nana Lv, Chengyuan Tao, Yannan Ou, Jiabao Wang, Xuhui Deng, Hongjun Liu, Zongzhuan Shen, Rong Li, Qirong Shen

**Affiliations:** a Jiangsu Provincial Key Lab of Solid Organic Waste Utilization, Jiangsu Collaborative Innovation Center of Solid Organic Wastes, Educational Ministry Engineering Center of Resource-saving fertilizers, The Key Laboratory of Plant Immunity, Joint International Research Laboratory of Soil Health, Nanjing Agricultural University, Nanjing, Jiangsu, People’s Republic of China; b The Sanya Institute of the Nanjing Agricultural University, Sanya, Hainan, People’s Republic of China; Agroscope

**Keywords:** soil-borne disease, disease suppression, soil microorganisms, endophytic microbiome, biocontrol agent

## Abstract

Members of the microbiotas colonizing the plant endophytic compartments and the surrounding bulk and rhizosphere soil play an important role in determining plant health. However, the relative contributions of the soil and endophytic microbiomes and their mechanistic roles in achieving disease suppression remain elusive. To disentangle the relative importance of the different microbiomes in the various plant compartments in inhibiting pathogen infection, we conducted a field experiment to track changes in the composition of microbial communities in bulk and rhizosphere soil and of root endophytes and leaf endosphere collected from bananas planted on Fusarium-infested orchards in disease-suppressive and disease-conducive soils. We found that the rhizosphere and roots were the two dominant plant parts whose bacterial communities contributed to pathogen suppression. We further observed that Pseudomonas was potentially a key organism acting as a pathogen antagonist, as illustrated by microbial community composition and network analysis. Subsequently, culturable pathogen-antagonistic Pseudomonas strains were isolated, and their potential suppressive functions or possible antibiosis in terms of auxin or siderophore synthesis and phosphate solubilization were screened to analyze the mode of action of candidate disease-suppressive Pseudomonas strains. In a follow-up *in vivo* and greenhouse experiment, we revealed that microbial consortia of culturable Pseudomonas strains P8 and S25 (or S36), isolated from banana plantlet rhizosphere and roots, respectively, significantly suppressed the survival of pathogens in the soil, manipulated the soil microbiome, and stimulated indigenous beneficial microbes. Overall, our study demonstrated that root-associated microbiomes, especially the antagonistic Pseudomonas sp. components, contribute markedly to soil suppression of banana Fusarium wilt.

**IMPORTANCE** Soil suppression of Fusarium wilt disease has been proven to be linked with the local microbial community. However, the contribution of endophytic microbes to disease suppression in wilt-suppressive soils remains unclear. Moreover, the key microbes involving in Fusarium wilt-suppressive soils and in the endophytic populations have not been fully characterized. In this study, we demonstrate that root-associated microbes play vitally important roles in disease suppression. Root-associated Pseudomonas consortia were recognized as a key component in inhibiting pathogen abundance associated with the host banana plants. This finding is crucial to developing alternate strategies for soilborne disease management by harnessing the plant microbiome.

## INTRODUCTION

Plant diseases can result in up to 20% annual losses of global food production (1). Soil-borne diseases severely hamper global crop production, as a range of pathogens can attack almost all cultivated agricultural crops, especially as the climate warms (2). Fusarium spp. are among the most devastating soilborne pathogens in crop production systems worldwide due to their extremely broad host range, causing diseases such as damping-off, root rot, and vascular wilt (3). The complex interactions of host plants with their associated microbes, which range from mutually beneficial to commensal to pathogenic, can significantly impact plant health and production (4). Therefore, understanding the contribution of plant-microbe interactions to soil disease suppression is a prerequisite for managing soilborne crop diseases.

Soil microorganisms play key roles in plant productivity and health through direct or indirect mechanisms, i.e., by mineralizing soil organic matter, activating plant defense mechanisms, and even producing antibiotics against phytopathogens (5–7). The rhizosphere microbiome, known as the second genome of plants, is recognized as the first line of defense against soilborne pathogens (8). Recent studies have shown that the endophytic microbial communities inhabiting plant compartments, such as the root (9) and the leaf endosphere (10), also contribute to plant growth and health, although the relative contributions of the individual communities still have to be confirmed. Thus, distinguishing the relative importance of the various plant-associated soil and endophytic microbial communities and understanding their respective modes of action are important steps toward designing and optimizing strategies for effective management of soilborne plant pathogens.

A rapidly growing body of literature has documented that the bulk soil is the main reservoir of microorganisms colonizing the rhizosphere (11, 12), so that the rhizosphere microbial community is a subset of the bulk soil microbes that are subsequently filtered by niche utilization attributes and interactions with the plant to inhabit the rhizosphere compartment (13). Meanwhile, a range of microbes with diverse functions may have adapted to and migrated into the plants and become endophytes that could be either candidate symbionts or stealth pathogens (14, 15). The microbiome of the leaf endosphere was recently found to be linked with soil suppression of soilborne disease by modifying plant exudates (16). The leaf endosphere microbiota is usually determined by spatial and temporal environments, genetic traits of the plant, disease-induced changes in plant leaves (17), and geographic location under certain conditions (18). At present, how the soil microbiome shapes the assembly of rhizosphere and endophytic microbiomes and their links to disease suppression remain poorly understood.

Disease-suppressive soil is a specific type of soil formed by plant-soil-microorganism interactions, whereby disease severity or incidence is maintained at a low level, even in the presence of the pathogen, susceptible host plants, and climatic conditions favorable for disease development (19). Soils that are suppressive to specific soilborne pathogens have been identified for a range of crops across many locations (20). Much effort has been invested in identifying key beneficial microbes (such as species or isolates of *Streptomyces*, Pseudomonas, *Flavobacterium*, etc.) with disease-suppressive traits that inhabit rhizosphere soils suppressive to various pathogens (21–23). Recently, there has been a growing interest in exploring the microbial composition of endophytic plant compartments from disease-suppressive fields (9). Despite growing awareness that endophytic microbiome interactions have an immense capacity to affect soil ecological functioning, it is still not clear how the endophytic microbiome influence the ability of a pathogen to become established in the soil.

The aims of the current study were to analyze the characteristics of bacterial communities and to identify the key species in the bulk and rhizosphere soils and within the endophytic compartments of roots and leaves from Cavendish banana plants cultivated in soils conducive or suppressive to Fusarium wilt (Panama disease), mainly caused by Fusarium oxysporum f. sp. *cubense* Tropical Race 4 (*Foc* TR4). To this end, we carried out a field experiment to monitor the changes in bacterial communities in soil (bulk and rhizosphere) and endophytic compartments corresponding to disease suppression. Furthermore, follow-up microcosm and mesocosm experiments were used to focus in on the impacts of the potentially beneficial microbes on disease suppression. By exploring the effects of soil microbiome communities on the assembly of endophytic microbiomes, we sought to achieve the necessary understanding required to harness the plant microbiome in order to predict and to enhance plant disease suppression.

## RESULTS

### Pathogen abundance.

**FIG 1:**
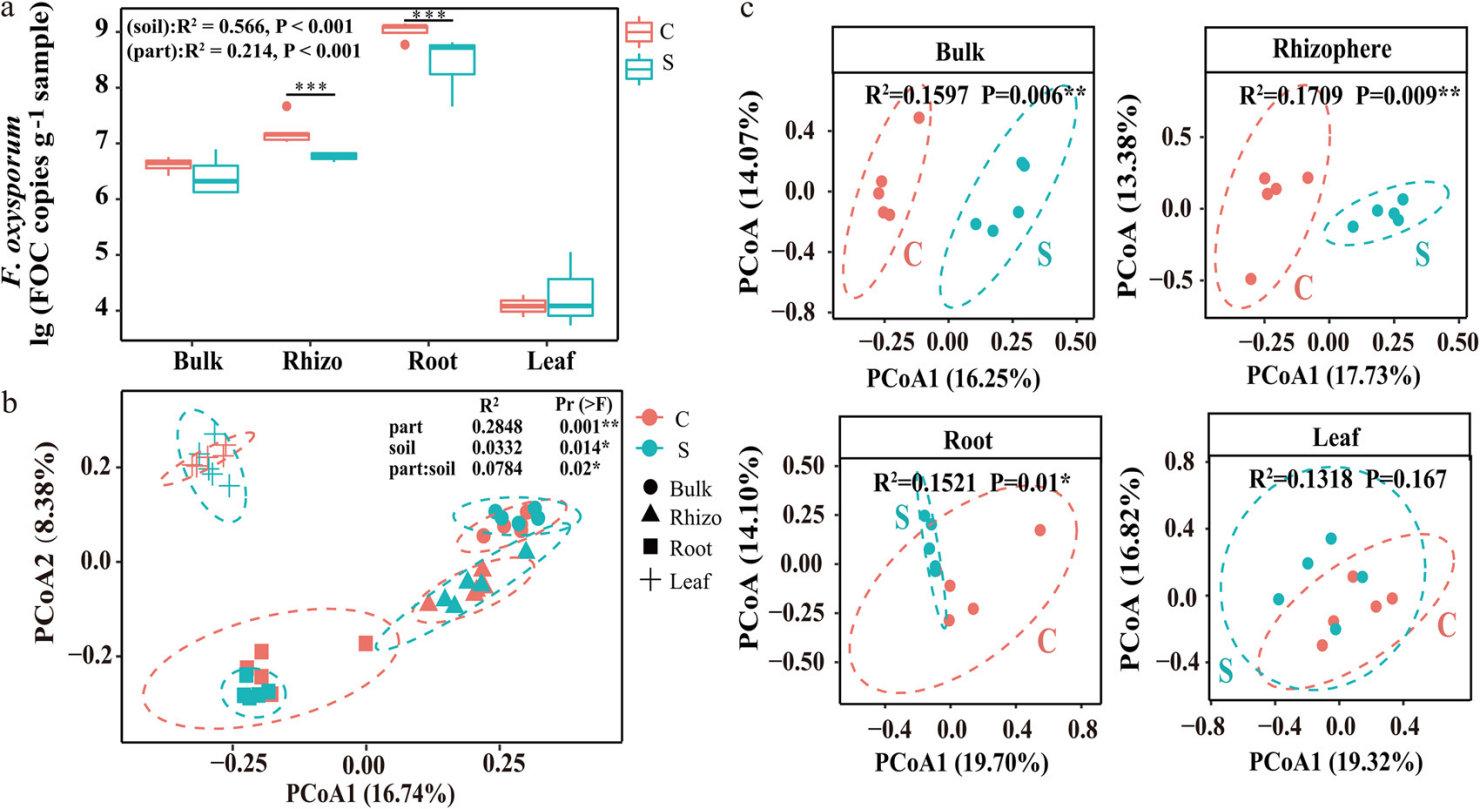
(a) Abundance of F. oxysporum in samples of disease-conducive banana orchard soil (C) and disease-suppressive banana orchard soil (S) (*t* test; means and standard deviations [SD]; *n* = 5; *, *P* < 0.01). (b) PCoA of whole bacterial communities across all samples. *P* values were determined by PERMANOVA. (c) PCoA of different parts of the community composition across all bacterial communities selected for the two banana orchard soil samples. *P* values were determined by ANOSIM.

The frequency of F. oxysporum as determined by quantitative real-time PCR (qPCR) was found to be highest in the roots, followed by the rhizosphere (Fig. 1a). Among the different compartments, the abundance was significantly lower in the rhizosphere soils and roots in banana plantlets grown in disease-suppressive soils than in those grown in disease-conducive soils (Fig. 1a).

### Bacterial community diversity and composition in disease-suppressive and -conducive soils.

For the Illumina MiSeq sequencing raw data, a total of 1,283,260 sequences for the 16S rRNA genes were retained after basic quality control. Analysis of bacterial alpha diversity indexes showed no significant difference for alpha diversity in the same compartment between disease-conducive and -suppressive soils, in spite of significant differences among different compartments being observed (see Fig. S1 in the supplemental material). Principal-coordinate analysis (PCoA) of the bacterial community compositions by permutational multivariate analysis of variance (PERMANOVA) revealed that bacterial composition was significantly different among the various compartments and between disease-conducive and disease-suppressive soils (Fig. 1b). Furthermore, the PCoA based on each compartment of banana plantlets grown on disease-conducive and disease-suppressive soils clearly showed significant differences with respect to bacterial communities in plants from disease-conducive and disease-suppressive soils in the compartments bulk soil, rhizosphere soil, and roots, although no significant difference was found in the leaf compartment (Fig. 1c).

**FIG 2:**
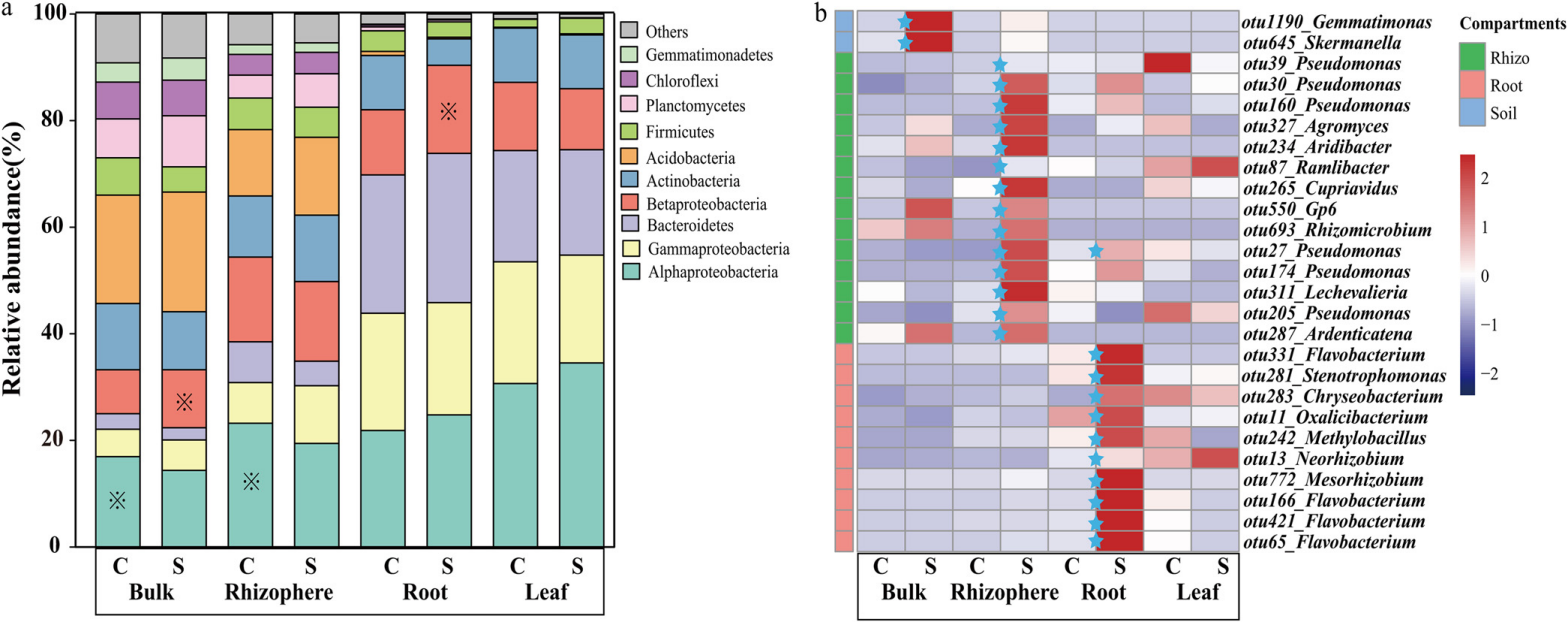
(a) Relative abundance of bacterial phyla for each treatment. “Others” indicates phyla with an extremely low abundance. C and S, growth in disease-conducive and disease-suppressive banana orchard soil, respectively. (b) Heat map showing Spearman’s rank correlation coefficients between bacterial OTUs and abundance of F. oxysporum in different compartments. Blue stars indicate that the relative abundance of the pathogen in the disease-suppressive soil is significantly higher than that in the disease-conducive soil.

Among the 1,283,260 high-quality sequences, 1,016,990 sequences (79.3%) were classified as bacteria, among which *Proteobacteria*, *Bacteroidetes*, *Acidobacteria*, *Actinobacteria*, and *Firmicutes* were the five most abundant phyla (Fig. 2a). The relative abundance of *Alphaproteobacteria*, *Gammaproteobacteria*, and *Bacteroidetes* gradually increased in the order bulk soil < rhizosphere soil < roots and leaves. Following analysis based on unpaired *t* tests, the relative abundance of *Alphaproteobacteria* was found to be significantly lower in both the bulk and the rhizosphere soils in the disease-suppressive soils than in the disease-conducive soils. Conversely, the relative abundance of *Betaproteobacteria* was found to be significantly higher in the compartments bulk soil and roots in the disease-suppressive soil than in the disease-conducive soil (Fig. 2a).

At a finer resolution, correlation analyses between the relative abundance of bacterial operational taxonomic units (OTUs) and the pathogen abundance in corresponding plant compartments showed that the relative abundance of a total of 42 OTUs was significantly and negatively correlated with pathogen abundance. Among these, the abundance of 26 OTUs was found to be significantly enriched in plants grown in the disease-suppressive soil in at least one of the compartments (Fig. 2b). Of these 26 OTUs, six (OTU30, OTU39, OTU160, OTU27, OTU174, and OTU205) belonged to Pseudomonas, which proved to be the dominant genus among these 26 potential key strains.

### Bacterial community network with the pathogen.

**FIG 3:**
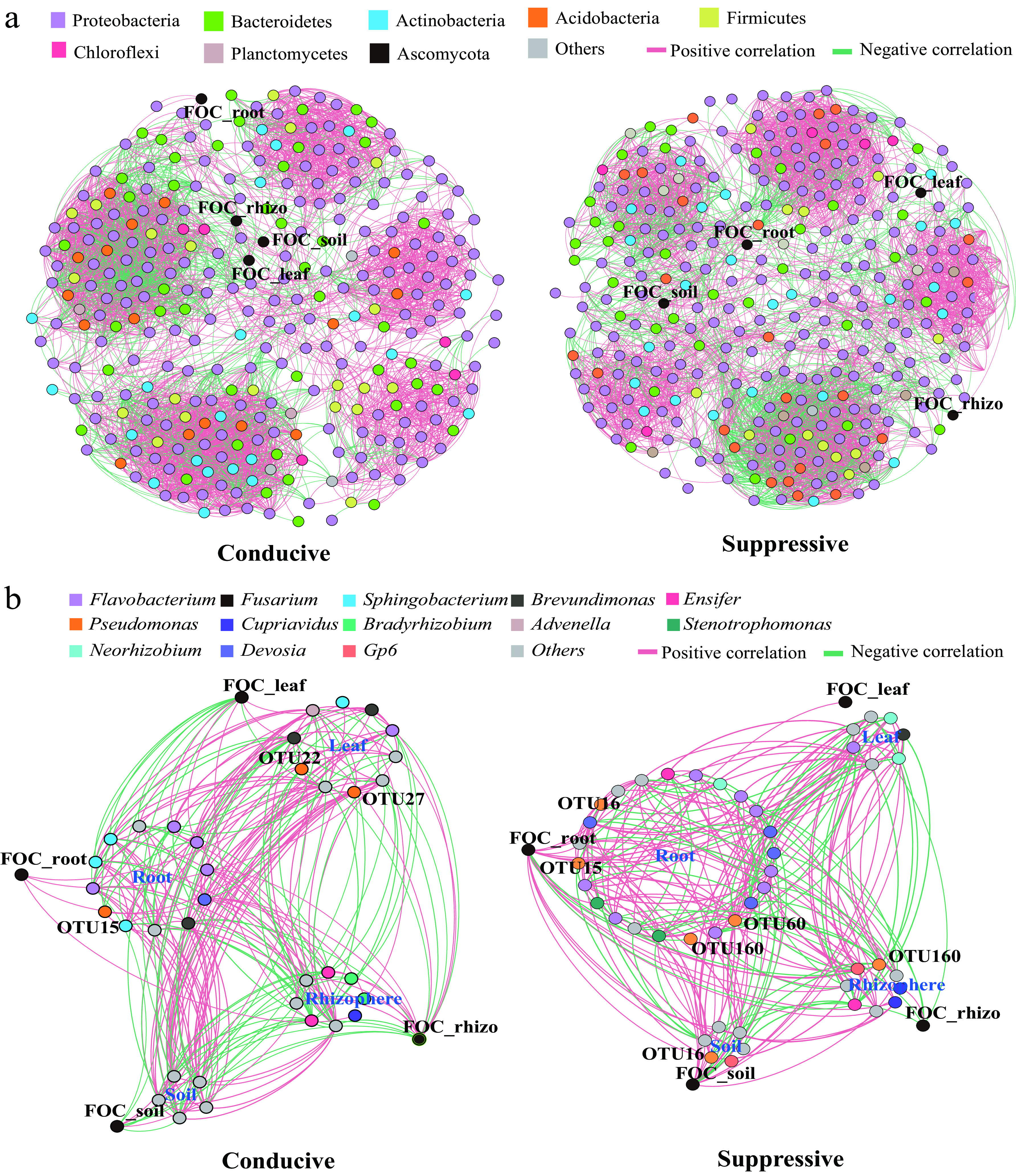
(a) Co-occurrence network of pathogenic F. oxysporum and bacterial communities within each compartment of banana plantlets grown in disease-conducive or disease-suppressive soil. Node colors indicate different bacterial phyla. (b) Subnetwork of pathogenic F. oxysporum and directly related microbial members. Node colors indicate different bacterial genera. Red edges represent positive correlations, and blue edges represent negative correlations.

The bacterium-pathogen ecological networks constructed for disease-suppressive and disease-conducive soils are illustrated in Fig. 3a, and the topological properties of the networks are shown in Table 1. The complex interactions between the disease-suppressive bacterial network and the pathogen network did not clearly differ, because the nodes and links were similar in the two networks. The negative relationship between two nodes was recognized as being because two closely related microorganisms may have mutually antagonistic interactions and showed that there were more potential antagonistic interactions among the bacterial community and the pathogen in disease-suppressive soils than in disease-conducive soils, since the proportion of negatively correlated links was 42.5% in the disease-suppressive network, compared with only 22.4% in the disease-conducive network.

**TABLE 1:** Topological properties of the microbial co-occurrence networks among different treatments^*a*^

Network	Total no. of:		Proportion of negative links (%)	Avg clustering coefficient	Modularity	Proportion of negative links (%) in compartment			
	Links	Nodes				Soil	Rhizosphere	Root	Leaf
Conducive	4,644	309	22.4	0.654	0.6887	0.311	0.203	0.156	0.449
Suppressive	4,649	331	42.5	0.626	0.6819	0.358	0.227	0.222	0.439

a The nodes and links were used to assess network complexity. The clustering coefficient shows the extent to which a node is connected to its neighbors. Modularity refers to the characteristics of the modules in the molecular ecological network.

**FIG 4:**
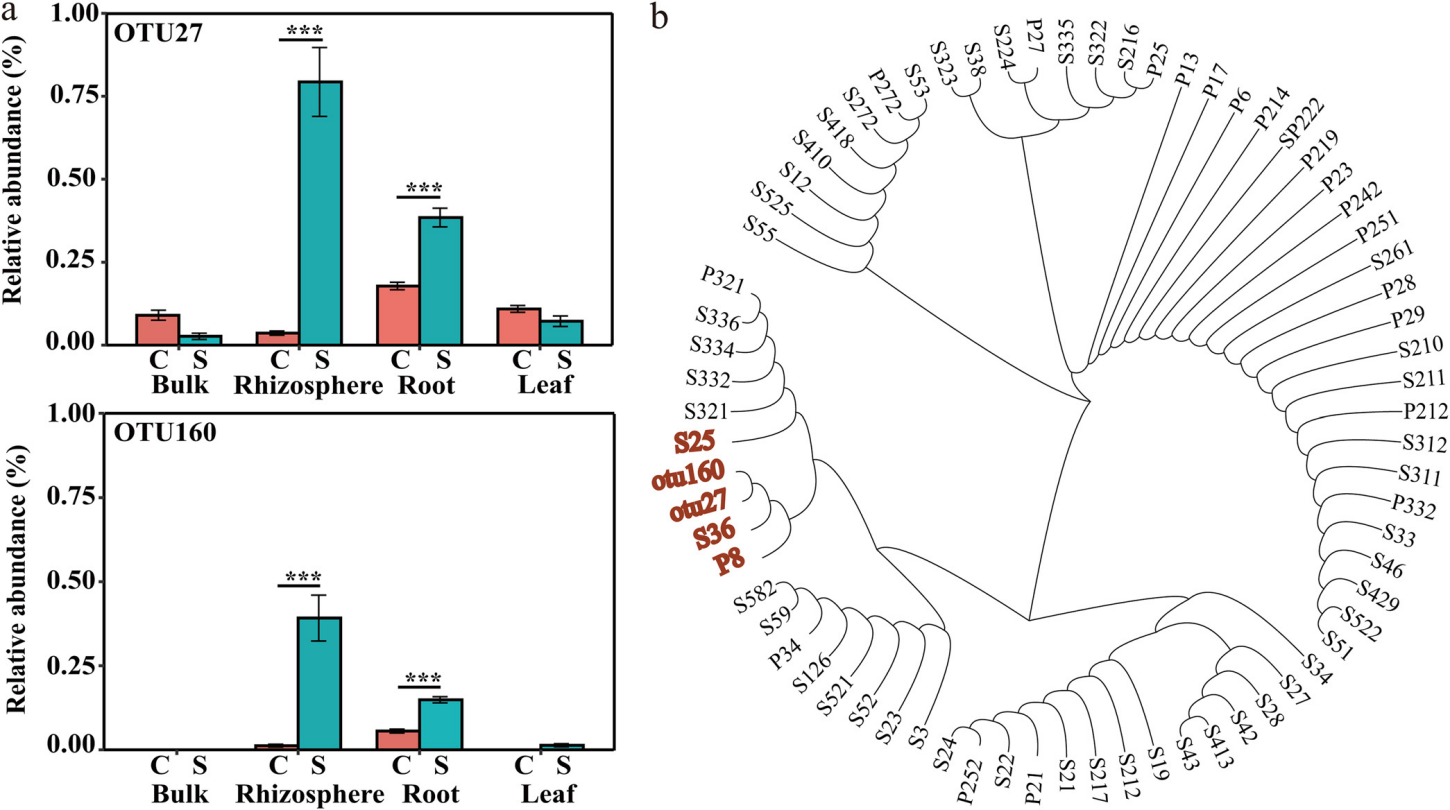
(a) Relative abundance of OTU27 and OTU160 in different banana plantlet compartments (Kruskal-Wallis rank sum test; means and SD; *n* = 5; *, *P* < 0.05) (b) Neighbor-joining phylogenetic tree constructed on the basis of the 16S rRNA gene sequences.

To more precisely characterize the key bacteria involved in controlling the pathogen abundance, subnetworks of the interactions among the pathogen and the bacterial community members were extracted and reconstructed from the disease-suppressive and disease-conducive networks (Fig. 3b). Interestingly, both the rhizospheric and the root endophytic bacteria, but especially the root bacteria, interacted most closely with the pathogen in the two subnetworks. More nodes (24 in the disease-suppressive soil and 12 in the disease-conducive soil) and links (94 in the disease-suppressive soil and 82 in the disease-conducive soil), especially in the root module, were observed in the disease-suppressive subnetwork than in the disease-conducive subnetwork. Moreover, bacteria of the genera *Devosia*, *Flavobacterium*, and Pseudomonas were commonly directly linked to the pathogen abundance in both reconstructed subnetworks. In both subnetworks, the abundance of the pathogen in the bulk soil, rhizosphere soil, and roots was negatively correlated with the abundance of Pseudomonas species. The relative abundance of OTU160, which was affiliated with Pseudomonas, was negatively correlated with pathogen abundance both in the rhizosphere soil and the roots in the reconstructed disease-suppressive subnetwork. Specifically, the relative abundances of OTU27 and OTU160, both affiliated with Pseudomonas, were significantly enriched in both the rhizosphere soil and the roots in the disease-suppressive soil compared with the disease-conducive soil (Fig. 4a).

### Culturable Pseudomonas isolates and their functional traits *in vitro*.

A total of 69 Pseudomonas strains were successfully isolated and identified from rhizosphere soil and roots in disease-suppressive soil. Among the *Foc* TR4-antagonistic Pseudomonas strains isolated, strains S25, P8, and S36 were phylogenetically most closely related to the potential key species of Pseudomonas OTU27 and OTU160 identified by sequencing analysis in this study (Fig. 4b).

**FIG 5:**
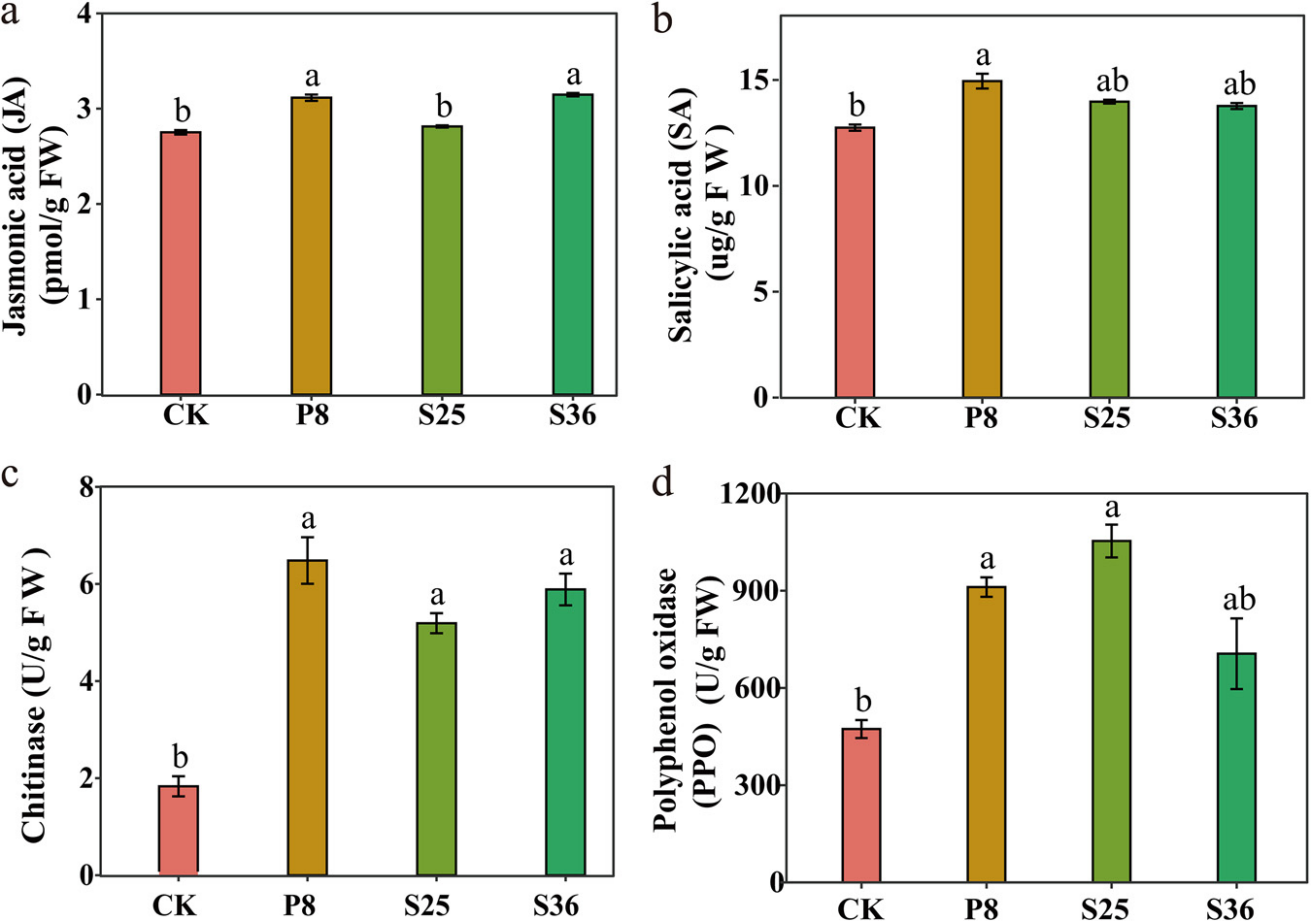
Physiological enzymatic activity in banana plants in the pot experiment with Pseudomonas inoculation (means and standard errors [SE]). (a) JA; (b) SA; (c) CHT; (d) PPO. Different letters above the bars indicate significant differences at the 0.05 probability level according to Duncan’s multiple-range test (*n* = 6). CK, control (treatment without Pseudomonas).

In order to further understand the effects of Pseudomonas on banana’s physiological and biochemical processes, we conducted a pot experiment to explore the effects of Pseudomonas sp. inoculation on plant hormones and physiological enzyme activities in banana plants. Our results showed that Pseudomonas P8 treatment displayed a significantly higher content of jasmonic acid (JA) and salicylic acid (SA) than the control (Fig. 5). Meanwhile, the activities of chitinase and polyphenol oxidase (PPO) were higher in the treatments treated with Pseudomonas P8 or S25 than the control.

**FIG 6:**
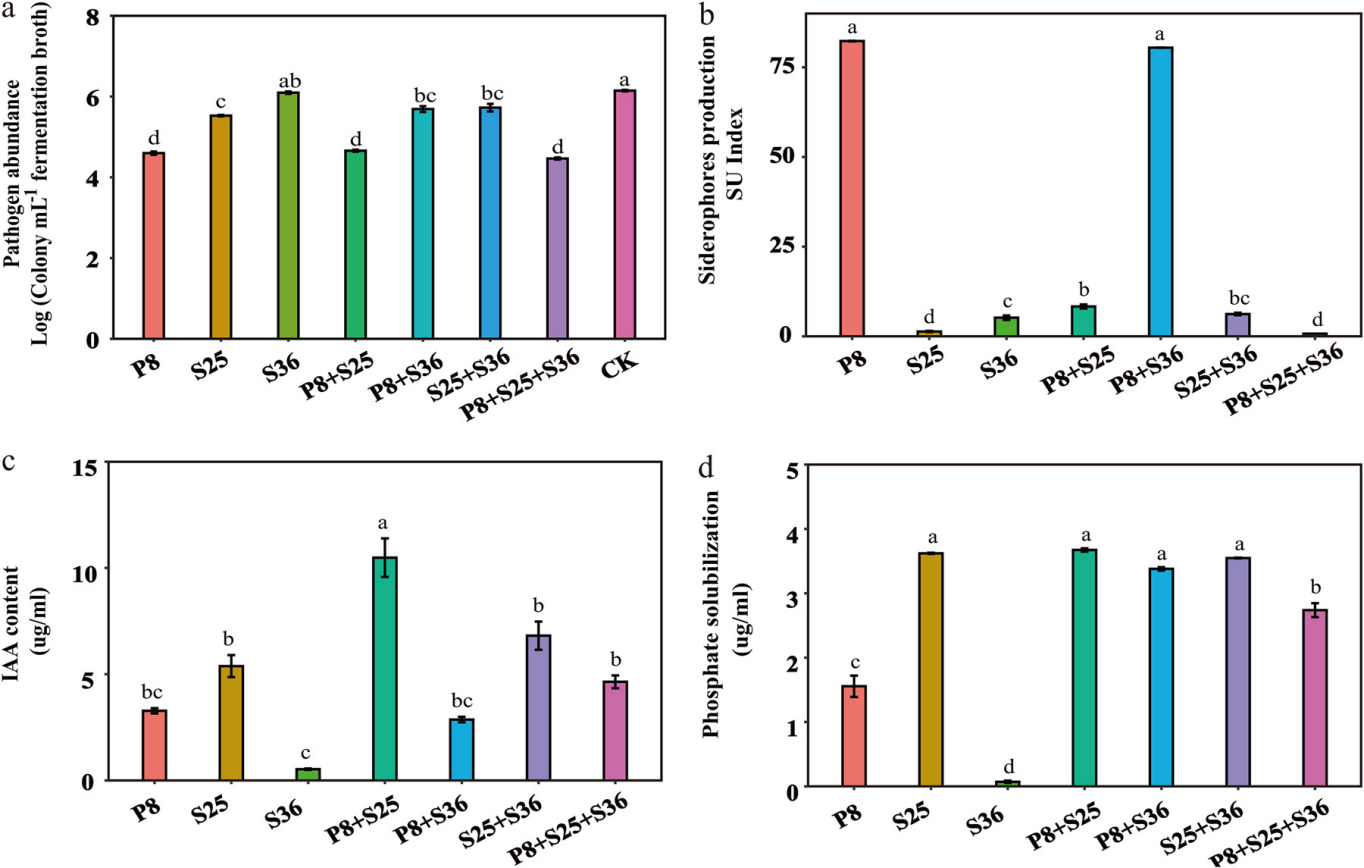
(a) Abundance of cultivable *Foc* TR4 in coculture of Pseudomonas strains and pathogen system. (b) Production of siderophores. (c) Production of auxin (IAA). (d) Solubilization of phosphate. P8, S25, and S36, treatments with single Pseudomonas strains; P8+S25, P8+S3, S25+S36, and P8+S25+S36, treatments with cocultures of Pseudomonas strains. Different letters above the bars indicate significant differences at the 0.05 probability level according to Duncan’s multiple-range test (*n* = 3). CK, control (treatment with *Foc* TR4 but without Pseudomonas).

Further analysis of the ability to antagonize the pathogen *in vitro* showed that strains P8 and S25 exhibited significant inhibitory effects on the pathogen, whereas strain S36 was not antagonistic to *Foc* TR4, as revealed by coculturing individual strains with the pathogen (Fig. 6a). However, P8, S25, their coculture (P8+S25), and cocultures with S36 (i.e., P8+S36, S25+S36, and P8+S25+S36) all showed significant antagonistic effects against the pathogen. Assays of siderophore biosynthesis showed that strain P8 and its coculture with S36 (P8+S36) showed a significantly higher ability to synthesize siderophores than did the other strains and their cocultures (Fig. 6b). The P8+S25 coculture exhibited the highest ability to produce indole-3-acetic acid (IAA), followed by S25+S36 (Fig. 6c). Strains S36, P8, and S25 and their cocultures all showed significant phosphorus-solubilizing activities (Fig. 6d).

### Pathogen-inhibitory ability of selected Pseudomonas strains in soil.

**FIG 7:**
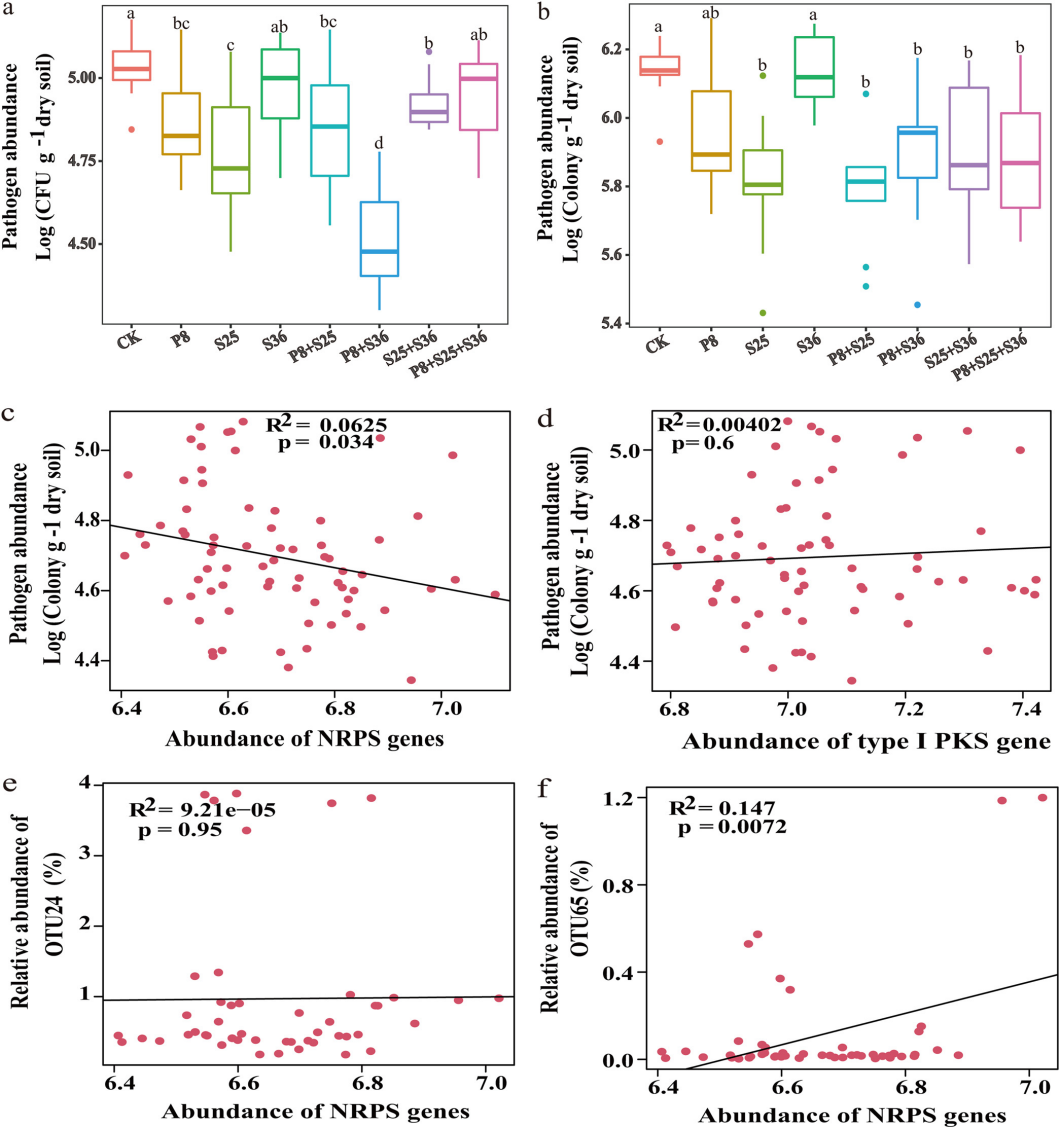
(a) Abundance of cultivable F. oxysporum f. sp. *cubense* (*Foc*) in the pot rhizosphere soil. (b) Abundance of F. oxysporum, determined by qPCR, in the pot rhizosphere soil. P8, S25, and S36, treatment with single strains of Pseudomonas; P8+S25, P8+S36, S25+S36, and P8+S25+S36, treatment with cocultures of Pseudomonas strains. Different letters above the bars indicate significant differences at the 0.05 probability level according to Duncan’s multiple-range test (*n* = 3). CK, control (treatment with *Foc* TR4 but without Pseudomonas). (c) Scatter diagram showing linear regression between the abundances of NRPS and pathogen density. (d) Scatter diagram showing linear regression between the abundances of type I PKS genes and pathogen density. (e) Scatter diagram showing linear regression between the abundances of NRPS genes and Pseudomonas OTU24 relative abundance greater than 0.1%. (f) Scatter diagram showing linear regression between the abundances of NRPS genes and Pseudomonas OTU65 relative abundance greater than 0.1%. The *r* and *P* values were calculated through Spearman correlation.

The greenhouse experiment results showed that after Pseudomonas inoculation, treatment with P8+S25 and S25+S36 exhibited a significantly improved dry weight of banana plants compared to the other treatments (Fig. S2). The greenhouse experiment showed that, compared with control (CK) soil, in which no inoculation with Pseudomonas strains took place, the numbers of culturable *Foc* TR4 organisms in all rhizosphere soil samples amended with antagonistic Pseudomonas strains or their cocultures were significantly reduced (Fig. 7a). This finding was broadly supported by real-time qPCR analyses (Fig. 7b). Consistent with the results for coculture of Pseudomonas and the pathogen, the pathogen in the soil amended with P8+S25 or P8+S36 exhibited a significantly lower population than that in the other soil treatments, as revealed by both culturable-colony counting and qPCR methods.

In order to explore the potential ability to produce antimicrobial compounds, the qPCR method targeting the nonribosomal peptide synthetase (NRPS) and type I polyketide synthase (PKS) genes was used to quantify their potential role in disease suppression after the inoculation of bacterial isolates. The result showed that the NRPS genes were significantly enriched in the banana rhizosphere after the inoculation of Pseudomonas-treated samples (S25, S25+S36, P8+S25, and P8+S25+S36), while the type I PKS genes were enriched only upon Pseudomonas P8 inoculation treatment (Fig. S3). Furthermore, the NRPS gene density, but not the type I PKS gene density, was significantly and negatively correlated with the abundance of pathogens (Fig. 7c and d).

**FIG 8:**
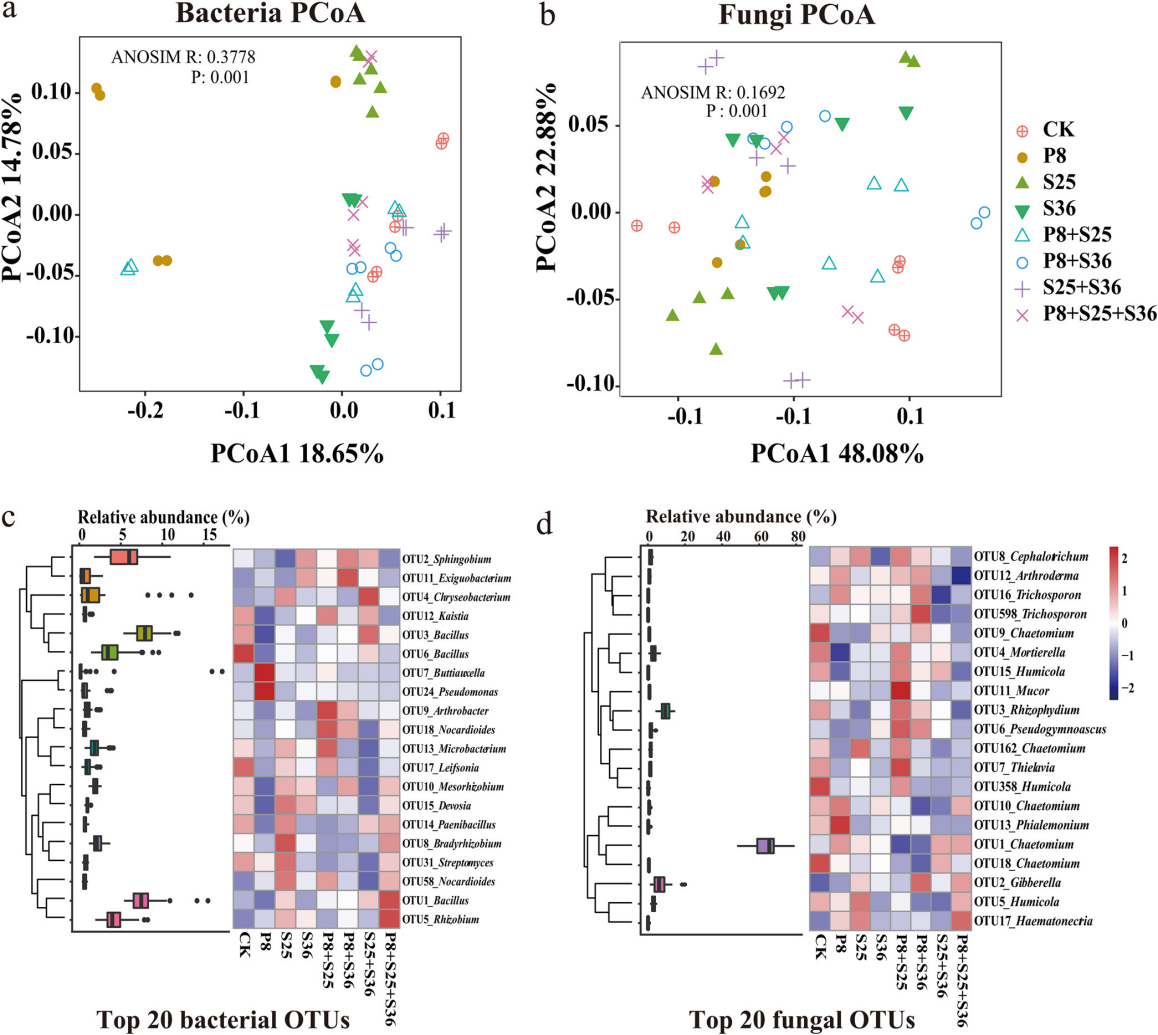
PCoA of the bacterial community (a) and fungal community (b) based on the Bray-Curtis distance for different treatments. The *P* values were calculated by ANOSIM. (c and d) The heat maps show the 20 most relatively abundant bacterial (c) and fungal (d) OTUs. The box diagrams indicate their relative abundances. The heat map scale ranges from −2 to 2 (blue to red).

Subsequently, results of the rhizosphere bacterial and fungal community sequencing showed that there were clear differences in microbial community composition according to the PCoA based on Bray-Curtis distances among different Pseudomonas strains (Fig. 8a and b). Further taxonomic analysis showed that the relative abundance of top 20 bacterial and fungal OTUs varied greatly across different Pseudomonas inoculation treatments (Fig. 8c and d). The inoculation of Pseudomonas spp. stimulated indigenous beneficial microbes, such as *Bacillus*, *Streptomyces*, and *Rhizobium*. Furthermore, relative abundances of Pseudomonas OTUs were higher in Pseudomonas inoculation treatments, especially Pseudomonas P8 inoculation (Fig. S4). Interestingly, the relative abundance of Pseudomonas OTU65 displayed a significant and positive correlation with NRPS gene density but not type I PKS gene density (Fig. 7f).

## DISCUSSION

In this study, banana plants without obvious Fusarium wilt symptoms from orchards with either disease-suppressive or disease-conducive soil were selected to investigate the pathogen abundance and bacterial diversity in the different compartments of banana, such as bulk and rhizosphere soils, roots, and leaves. Our aim was to characterize the relative contributions to disease suppression of individual members of the bacterial community in the different plant compartments in disease-suppressive soils. Rhizosphere soil and roots were found to be the most important compartments for hosting bacteria to achieve pathogen suppression, since the pathogen abundance and bacterial composition in these compartments differed significantly. Pseudomonas was identified as a potential key taxon to inhibit the soilborne pathogen in the rhizosphere soil and root of banana grown in the disease-suppressive orchard. Furthermore, culturable Pseudomonas strains were isolated, and their functional traits, which could contribute to soilborne disease suppression, was demonstrated by *in vitro* and *in vivo* experiments.

Soil pathogen abundance is a sensitive indicator that can be used in determining soil suppression of Fusarium wilt disease (24). Supporting previous reports that disease-suppressive soils usually harbor a lower pathogen abundance than disease-conducive soils (25, 26), a lower abundance of F. oxysporum was found in the current study in the rhizosphere of plants grown in the disease-suppressive soil than in that of plants in the disease-conducive soil. In agreement with a previous finding that a reduction of pathogen abundance was observed in the roots of cucumber planted in a disease-suppressive soil (26), a lower abundance of pathogen was also found in the present study in banana roots grown in soil suppressive to Fusarium wilt disease. These results together suggest that the pathogen abundance in the rhizosphere and root are the most important targets to distinguish between disease-suppressive and disease-conducive conditions.

Both PCoA and comparison of the bacterium-pathogen interaction networks revealed that, regardless of the disease-suppressive and disease-conducive conditions, the rhizosphere and the roots (and the bacteria therein) are the compartments most closely interacting with the pathogen. Our results are in line with a large number of previous reports demonstrating the importance of rhizosphere microorganisms in achieving plant disease suppression (22, 27, 28). The rhizosphere is the environment where the beneficial and pathogenic microorganisms exert the greatest influence on plant health and growth (29, 30). Hence, the rhizosphere microbiome could contribute to securing plant health, probably via a range of plant physiological and biochemical activities, e.g., plant nutrient acquisition and hormonal homeostasis (4, 31). Consistent with previous reports that the root microbiome plays an important role in suppressing the pathogen (9), the roots of tissue-cultured banana plantlets grown in disease-suppressive soil were found to have assembled a bacterial community distinct from that in plantlets planted in disease-conducive soil, indicating that the root microbiome is associated with antagonism against the pathogen. These results demonstrate that the banana rhizosphere and root endophytic microbiomes play critical roles in disease-suppressive soils.

The compositions of the rhizosphere microbiome communities have been widely reported to be mainly determined by the soil type and the plant genotype (11). In the present study, the composition of the banana rhizosphere microbiome was also affected by the microbial community in the bulk soil, as revealed by PCoA, supporting the previous conclusion that soil is the “seed bank” of plant endophytes (4). In broad agreement with the previous finding that the composition and structure of microbial communities in grapevine soil and different compartments of grapevines are significantly different (32), clearly different bacterial communities in banana roots were found between plantlets grown in disease-suppressive and disease-conducive soils. Thus, our results suggest that banana grown in the disease-suppressive soil could assemble a distinctive root microbiome which can prevent pathogen invasion.

In this study, *Proteobacteria*, *Bacteroidetes*, *Actinobacteria*, *Actinobacteria*, and *Firmicutes* were the most abundant phyla, exhibiting great variation in relative abundance across different plant compartments and between disease-suppressive and disease-conducive soils. Similarly, these phyla were widely found to be the dominant bacterial groups in the various soils and plant endospheres (32). The relative abundance of *Proteobacteria* increased substantially in the plant root and leaf endosphere. This result corroborates previous findings which showed that *Proteobacteria* dominated in the plant endosphere, including roots and leaves (33). Furthermore, *Betaproteobacteria* was significantly enriched in the bulk soil and roots of plantlets grown in the disease-suppressive soil relative to those of plantlets in the disease-conducive soil, a finding which is in accordance with a previous report that *Betaproteobacteria* abundance was linked with suppression of damping-off disease caused by the soilborne fungal pathogen Rhizoctonia solani (22). Although no significant difference in *Gammaproteobacteria* was found, the results of comparison of the relative abundance of OTUs between disease-suppressive and disease-conducive soils together with the results of the correlations between OTUs with pathogen abundance illustrated that most of the potential key OTUs related to disease suppression were species of Pseudomonas, a keystone genus that belongs to *Gammaproteobacteria* and has been found in soils suppressive to banana Fusarium wilt (34), wheat take-all disease (35, 36), and black root rot (37).

The primary mechanism of action ascribed to *Gammaproteobacteria* populations for suppressing diseases is related to biological activity of antibiotics. Many bacterial species belonging to the genus Pseudomonas produce high concentrations of secondary metabolites that act either as antibiotics or as potential biocontrol agents (BCAs) (38, 39). Fluorescent strains of Pseudomonas producing antibiotics are currently used in biological treatments for enhancing soil suppressiveness (40). In addition, several other OTUs affiliated with *Flavobacterium* and *Rhizomicrobium* were also found to be key candidate groups involved in disease suppression in the current study. This result was in line with previous findings that identified *Flavobacterium* and *Rhizomicrobium* as widely recognized plant-growth-promoting rhizobacteria or keystone species acting in the suppression of soilborne pathogens (41, 42). Altogether, our results illustrate that beneficial bacteria dominated in the rhizosphere and root microbiomes in banana planted in disease-suppressive soils and contributed to suppression of banana Fusarium wilt.

Given that OTUs classified as Pseudomonas dominated among the potential key species and were identified as key members of the network suppressing the pathogen, culturable Pseudomonas strains were isolated and screened in detail. In this study, OTUs affiliated with Pseudomonas were observed in all soil and plant compartments, indicating that Pseudomonas is an indigenous genus in the plant endosphere, rhizosphere, and leaf endosphere, most isolates of which are established as symbiotic bacteria in these niches (43). Pseudomonas populations have regularly been shown to be involved in the suppression of Fusarium wilt disease or wheat take-all disease (37, 44), as well as playing a vital role in inhibiting infection by pathogens in the banana endophytic microbiome (45). In the present study, the assay of three culturable Pseudomonas strains showed that two strains, P8 and S25, could significantly antagonize the pathogen of *Foc* TR4, whereas strain S36 exhibited no obvious effects in suppressing this pathogen; it had previously been reported that not all Pseudomonas spp. could directly suppress the pathogen (44). Coculture assays in our study demonstrated that cocultures of antagonistic Pseudomonas strains with other, even nonantagonistic Pseudomonas strains could support a stronger suppressiveness toward banana Fusarium wilt; previous studies had reported that probiotic diversity enhanced the suppressive ability of the rhizosphere microbiome against bacterial wilt disease (46, 47).

In this study, we were surprised to find that the defense system in Pseudomonas-treated banana plants was more responsive to the inoculation of Pseudomonas, as shown by the increases in jasmonic acid and salicylic acid contents and also the activities of chitinase and polyphenol oxidase compared to the control. Jasmonic acid and salicylic acid pathways are two major resistance-related signal pathways in plants (48–50) which promote the biosynthesis of stress resistance-related substances (51, 52). As chitin is the main component of the cell wall of plant-pathogenic fungi, chitinase can destroy the cell wall of such fungi by degrading chitin, thus killing them (53–55); this can be used to control plant diseases and pests. Our experimental results are consistent with previous studies showing that chitinase plays an important role in plant antifungal disease response (56, 57). Together, our results demonstrated that Pseudomonas inoculation enhanced the plant defense system against pathogens.

Further, we demonstrated a particular ability of Pseudomonas P8, and its cocultures with other selected Pseudomonas strains, to directly suppress Fusarium spp. in an *in vitro* assay. As competition for iron between beneficial bacteria and the pathogen can determine infection (58), the ability of the suppressive Pseudomonas strain P8 and its coculture with Pseudomonas S36 to produce more siderophores, as determined by the *in vitro* assay, indicates a possible mechanism by which pathogen suppression occurs. These results agree with previous findings that the potential mechanism of Pseudomonas achieving pathogen suppression was linked with secretion of antimicrobial compounds or by niche competition (22, 59). Although Pseudomonas P8 exhibited low levels of IAA production and phosphate solubilization, relative to the other two suppressive strains tested, the coculture of Pseudomonas P8 with strain S25 or especially with S36 significantly enhanced the potential functions in promoting plant growth. These results support another previous finding that the contribution of Pseudomonas to disease suppression could arise through indirect stimulation of plant immunity (20, 60). Our data indicate that the combined action of specifically antagonistic Pseudomonas strains (P8) together with other Pseudomonas strains (S25 and S36) leads to marked decreases in the pathogen abundance in the banana rhizosphere. In our study, not all cocultures of Pseudomonas species performed better than the monocultures in terms of disease suppression, and pathogen abundance was not systematically improved by the inclusion of any particular Pseudomonas strain. This result was in line with a previous finding that bacterial interspecific interactions can also be involved in plant disease management (61).

Moreover, it was reported that NRPS and PKS genes were important for plant health, especially when the hosts were infected by soilborne pathogens (62, 63). The NRPS gene can synthesize a great deal of secondary metabolites, for example, those serving as antimicrobial antibiotics in agricultural production (64, 65). Our experimental results showed that Pseudomonas inoculation stimulated enrichment of Pseudomonas and NRPS genes in soil, which is in accordance with previous studies showing that Pseudomonas is one of the richest taxa that contain biosynthetic genes for antibiotics (66, 67). In line with previous work (68), syntrophic interactions mediated by the exchange of nutrients and secondary metabolites might determine the functional traits of cocultures. However, a complete understanding of the interstrain interactions among Pseudomonas spp. requires further follow-up experiments in order to dissect the strain synergies in the rhizosphere context.

In summary, by comparing the composition of the microbial communities inhabiting different soil and plant compartments, we demonstrated that bacterial communities in the banana rhizosphere and roots are the two dominant niches with regard to pathogen suppression. We further observed that beneficial bacteria were enriched in these two compartments of banana plantlets grown in disease-suppressive soil, and Pseudomonas was identified by microbial composition and network analysis as a potentially key organism, acting as a pathogen antagonist. Follow-up *in vitro* and greenhouse experiments involving isolation of culturable antagonistic Pseudomonas strains and assaying their potential functions in auxin or siderophore synthesis and phosphate solubilization revealed that microbial consortia of culturable Pseudomonas strains P8 and S25 (or S36), isolated from the rhizosphere and roots of banana plantlets grown in disease-suppressive soils, significantly suppressed the pathogen abundance in the soil. Pseudomonas spp. with antagonistic ability and their combinations significantly increased the activity of disease-resistant enzymes in banana plants, stimulated the expression of NRPS genes, and manipulated microbial communities in rhizosphere soil. Together, our findings show that root-associated microbiomes, especially the antagonistic Pseudomonas spp., contribute to soil suppression of banana Fusarium wilt disease.

## MATERIALS AND METHODS

### Field experimental design.

The disease-suppressive and disease-conducive banana orchards used in this study were located at Fushan Town, Chengmai County, Hainan Province, China (109.92°E, 19.83°N). The two banana orchards were adjacent to one another in a typical, tropical monsoon climate with a mean annual temperature of about 23°C and a mean annual precipitation of 2,250 mm. Both orchards had been mono-cropped with banana (*Musa*, AAA Cavendish cv. Brazil) for more than 10 years. The Fusarium wilt disease incidences in the disease-suppressive and disease-conducive orchards were 7.3% and 66.8%, respectively, in 2016, according to the farm records. Basic soil physicochemical properties from these paired orchards are described in Table S1. In August 2016, all banana trees were cut down and soils were rotary ploughed for the subsequent field experiment. Both the disease-suppressive and the disease-conducive orchards were replanted with banana tissue-cultured plantlets at a density of approximately 1,950 plants per ha. The use of irrigation, fertilizers, and pesticides was the same for the disease-suppressive and disease-conducive orchards.

### Sample collection and processing.

Five representative replicate subplots of 50 by 40 m within each orchard were randomly selected for sample collection in July 2017 during the banana harvest stage. In each subplot, three banana plants without obvious wilt symptoms, at least 3 m apart, were selected for sampling of the surrounding soil (bulk and rhizosphere) and plant endophytic compartments (roots and leaves). For each tree, three soil cores under the drip line were randomly dug to a depth of 20 cm, using a shovel. Bulk soil with roots were collected by hand, with latex gloves, while a piece of leaf tissue (10 by 10 cm) was collected from the latest developed leaf from each plant as the leaf sample. All samples were transferred to the laboratory on ice and processed immediately. For each replicate subplot, nine soil cores were pooled, and 1 kg was subsampled and defined as the bulk soil.

All sampled roots were shaken gently to remove loosely adhering soil and were then used for rhizosphere soil collection, according to the previously described method (69). Briefly, soil tightly bound to the roots was recovered by rinsing with 0.9% (wt/wt) sterilized physiological saline solution (SPSS) and shaken at 170 rpm for 30 min at room temperature. Subsequently, the soil suspension was centrifuged at 10,000 × *g* for 10 min, with the resulting pellet being defined as rhizosphere soil. The roots and leaves were cut into 5-cm lengths, washed with sterile water, and transferred to a 2% (wt/wt) sodium hypochlorite solution to soak for 15 min to achieve surface sterilization. After being washed with sterile water, the banana tissues of each replicate sample were soaked again in 70% (wt/wt) ethanol solution for 5 min. All roots and leaves for each replicate were rinsed with sterile water again to remove the residual alcohol and then patted dry with sterile paper. Finally, all roots and leaves for each replicate were ground using a sterile porcelain mortar and pestle. All samples were divided into two parts: one part was mixed with 30% (vol/vol) glycerin and stored at −80°C for subsequent isolation of culturable bacteria, whereas the second part was used for genomic-DNA extraction.

### DNA extraction and pathogen determination.

The total genomic DNA of bulk soils, rhizosphere soils, roots, and leaves was extracted from a 0.5-g sample using the DNeasy PowerSoil kit (Qiagen GmbH, Hilden, Germany) according to the manufacturer’s instructions. The concentration and quality of the DNA were determined using a NanoDrop 2000 spectrophotometer (Thermo Scientific, Waltham, MA, USA).

The abundance of the pathogen causing Fusarium wilt disease (F. oxysporum f. sp. *cubense* Tropical Race 4 [*Foc* TR4]) in the surrounding bulk and rhizosphere soils, roots, and leaves was quantified using the extracted DNA template by the previously described real-time qPCR protocol (70). The PCR amplicons were conducted with the primers FOF1 (5′-ACA TAC CAC TTG CCT CG-3′) and FOR1 (5′-CGC CAA TCA ATT TGA GGA ACG-3′) using a 7500 real-time PCR system (Applied Biosystems, Foster City, CA, USA). Standard curves were generated by using 10-fold serial dilutions of a plasmid containing a fragment copy of the internal transcribed spacer (ITS) from *Foc* TR4. Each assay was performed in triplicate, and the results were expressed as log(target copy number/gram of sample) prior to further statistical analysis.

### Amplicon library construction and sequencing.

Bacterial sequencing libraries were constructed according to previously described protocols (71, 72). The gene-specific primers 520F (5′-AYT GGG YDT AAA GNG-3′) and 802R (5′-TAC NVG GGT ATC TAA TCC-3′) were used to amplify the V4 hypervariable region of bacterial 16S rRNA genes. The final library quality and concentration were determined with an Agilent 2100 Bioanalyzer instrument (Agilent Technologies Co. Ltd., California, USA). All libraries constructed were sequenced using the Illumina HiSeq 2500 platform at Guangdong Magigene Biotechnology Co., Ltd. (Guangzhou, China).

### Sequence data processing.

Raw sequences were assigned to individual samples according to the unique barcode, and the adapter and primer sequences were trimmed in QIIME and USEARCH (73). After removal of low-quality sequences, single-stranded sequences in the forward and reverse directions were merged. The merged sequences were then processed according to the UPARSE pipeline to generate OTUs (74). A representative sequence for each OTU was selected and classified using the RDP classifier against the RDP bacterial 16S database (75). OTUs affiliated with archaea and chloroplasts were removed from the OTU table. The relative abundance of each OTU was calculated as the number of sequences affiliated with the target OTU.

### Isolation and identification of culturable Pseudomonas strains and assay of pathogen inhibition *in vitro*.

Given the demonstrated role of Pseudomonas in banana Fusarium wilt disease suppression (34) and the results from bacterial community analyses, the isolation, identification and antagonistic ability of Pseudomonas against *Foc* TR4 were tracked by culture-dependent methods. The collected rhizosphere soils and roots from the disease-suppressive orchard, which had previously been mixed with glycerin and stored at −80°C, were used to isolate culturable Pseudomonas strains. After 10-fold serial dilutions were made, appropriate suspensions were spread onto King’s B medium (KB) and incubated at 30°C for 36 h. Isolated Pseudomonas strains were cultured in KB broth, and then genomic DNA was extracted using a rapid one-tube genomic DNA extraction protocol (76). The primers 27F (5′-AGA GTT TGA TCC TGG CTC AG-3′) and 1492R (5′-TAC GGT TAC CTT GTT ACG ACT T-3′) were used to amplify the bacterial 16S rRNA genes by using 16S rRNA gene PCR kits (TaKaRa, Dalian, China) to obtain the phylogenetic information. Moreover, the ability of Pseudomonas isolates to suppress the growth of *Foc* TR4 was tested *in vitro* by a dual-culture assay as previously described (77).

### Functional trait assays of selected Pseudomonas strains.

Based on the phylogenetic analysis result, the effects of inoculation of three selected Pseudomonas strains (P8, S25, and S36) on banana physiological processes were conducted. In brief, banana plants were grown for 5 weeks in sterilized substrate pots in a growth chamber. When the first leaf was fully expanded, Pseudomonas (S25, S36, and P8) inoculation was performed by injecting the soil with a water suspension containing 10^8^ cells mL^−1^ (as determined by optical density at 600 nm). Meanwhile, sterilized water was introduced as a control (CK). After inoculation for 48 h, the second leaf was collected for the physiological test. Leaves were frozen in liquid nitrogen immediately after harvest, finely powdered in liquid nitrogen using a mortar and pestle, and kept frozen until use. The leaf powder was extracted with 1 mL of the corresponding abrasive fluid buffer, and the supernatant was centrifuged according to the kit method of Suzhou Keming Biotechnology Co., Ltd., for colorimetric determination of jasmonic acid (JA), salicylic acid (SA), polyphenol oxidase (PPO), and chitinase (CHT).

Subsequently, three Pseudomonas strains (P8, S25, and S36) and their cocultures were selected for further potential functionality assays. Three probiotic traits which are potentially beneficial for plant growth promotion, namely, auxin (IAA) production, siderophore synthesis, and phosphate solubilization, as well as suppression of *Foc* TR4, were assessed. To measure IAA production, all Pseudomonas strains and cocultures were cultured in Luria-Bertani (LB) medium for 72 h at 30°C with shaking at 170 rpm. The concentration of IAA in the fermentation broth was then determined according to the Salkowski method (78, 79). To measure siderophore production, all probiotic Pseudomonas strains and cocultures were cultured in liquid modified King’s B (MKB) medium for 48 h at 30°C with shaking at 170 rpm (80). The siderophore concentration in the fermentation broth was then determined according to the published method (81). To measure phosphate solubilization, all Pseudomonas strains and cocultures were grown in National Botanical Research Institutes phosphate nutrient (NBRIP) medium (82) for 7 days at 30°C with shaking at 170 rpm. The phosphate solubilization ability was assessed by using the molybdenum antimony colorimetric method (83). To measure the antagonistic ability of the three selected Pseudomonas cocultures, the cocultures were grown in potato dextrose broth (PDB) medium with *Foc* TR4 for 5 days at 28°C with shaking at 170 rpm; glass beads were added to the medium to prevent the hyphae of the pathogen from caking. After 10-fold serial dilutions were made, appropriate suspensions were spread onto Martin medium and incubated for 36 h at 28°C to count the *Foc* TR4 organisms (84).

### Assay for the ability of selected Pseudomonas strains and cocultures to suppress *Foc* TR4 in soil.

The biocontrol efficiency of selected Pseudomonas strains and their cocultures against *Foc* TR4 in soil was assessed by a 90-day greenhouse experiment. The test soil was collected from the disease-conducive orchard under study, located at Fushan Town, Chengmai County, Hainan Province, China. After being passed through a 5-mm sieve, the soil was mixed with sterilized seedling substrate (Jiangsu Xing Nong Substrate and Technology Co., Ltd.) at a 1:9 (vol/vol) ratio. Seven treatments were set up: soil amended with three selected Pseudomonas strains (P8, S25, and S36) or their cocultures (P8+S25, P8+S36, S25+S36, and P8+S25+S36), plus a CK amended with 20-mL sterile LB broth. Each treatment consisted of six replicate pots, and each pot, which was loaded with 200 g collected soil, was planted with one tissue-cultured banana plantlet. At 60 days after planting, the banana plants were inoculated with 20 mL Pseudomonas fermentation broth or an equal volume of sterile LB broth, according to the experimental design, by the root drench method, with a final concentration of 5.0 × 10^7^ CFU of bacteria per g dry soil (85). Seven days after inoculation with Pseudomonas, the pathogen *Foc* TR4 was inoculated at a final concentration of 10^5^ CFU of bacteria per g soil. During the experimental period, banana plantlets were irrigated with sterile water when necessary. Pots with banana plantlets for each treatment were rearranged randomly every 2 days. Typical symptoms of Fusarium wilt, including leaf yellowing, pseudostem splitting, browning, and discoloration of vascular tissues were monitored daily following inoculation of *Foc* TR4 into the soil.

The rhizosphere soil samples were collected in June 2022, when all of the control bananas exhibited wilt symptoms. The rhizosphere soil was processed, and soil genomic DNA was extracted according to the method described above. The abundance of *Foc* TR4 was determined by the previously described method (70). The bacterial (72) and fungal (71) libraries for sequencing were constructed following protocols described above. Gene-specific primers (515F, 5′-GTG CCA GCM GCC GCG GTA A -3′; 907R, 5′-CCG TCA ATT CMT TTR AGT TT -3′) were used to amplify the V4-V5 region of bacterial 16S rRNA genes, while primers ITS1F (5′-CTT GGT CAT TTA GAG GAA GTA A-3′) and ITS2 (5′-GCTGCGTTCTTCATCGATGC-3′) were used to target the ITS1 region of the fungal ITS.

The abundances of the NRPS gene (A3F, 5′-GCS TAC SYS ATS TAC ACS TCS GG-3′; A7R, 5′-SAS GTC VCC SGT SCG GTA-3′) (86) and type I modular PKS gene (degKS2F, 5′-GCI ATG GAY CCI CAR CAR MG IVT -3′; degKS2R, 5′-GTI CCI GTI CCR TGI SCY TCI AC-3′) (87) were determined by using the qPCR method. These two primer pairs amplify a variety of biosynthetic gene clusters (BGCs) belonging to NRPS and type I PKS. Quantitative PCRs for NRPS and PKS were performed using the temperature profiles provided by Zhao et al. (88). Meanwhile, densities of culturable *Foc* TR4 in the rhizosphere soil were determined by using the serial dilution colony counting method.

### Bioinformatics analyses.

Statistical analyses were performed using the IBM SPSS 20.0 software program (IBM Corporation, New York, USA) and R software programs (version 3.5.0). Nonnormal data were log transformed when necessary or analyzed using nonparametric tests. All statistical tests performed in this study were considered significant at a *P* value of <0.05. Multiplicity analysis of variance, unpaired *t* tests, and one-way ANOVA (with Duncan’s multiple-range test) were performed to determine significant differences among treatments. PCoA based on the weighted UniFrac metric was performed to evaluate the differences in bacterial communities among different treatments using the vegan package in the R3.5.0 platform. PERMANOVA was performed to test for significant differences among various treatments using the vegan package.

Microbial alpha diversity indexes (Chao1 and Shannon) were calculated based on randomly resampled OTU abundance matrices at the same depth (8,483 sequences) in mothur (https://mothur.org). Linear discriminant analysis (LDA) effect size (LEfSe) analysis was performed to identify key bacterial taxa between disease-suppressive and disease-conducive soils (89). The nonparametric Kruskal-Wallis (KW) sum-rank test was used in LEfSe analyses to detect the features with significantly different abundances between assigned classes, and LDA was then performed to estimate the size of the effect of each differentially abundant taxon (89). Spearman’s rank correlation coefficients between the relative abundances of OTUs and *Foc* were calculated using R3.5.0 software. *P* value adjustments for multiple comparisons were performed using the false discovery rate (FDR) correction (90).

Potential ecological interactions among bacteria in surrounding soils (bulk and rhizosphere) and plant endophytic compartments (roots and leaves) were determined by modeling the microbial community using co-occurrence network analysis (91). The bacterial OTUs in the bulk soil, rhizosphere soil, roots, and leaves of plants from disease-suppressive and disease-conducive soils and the pathogen were pooled to construct the ecological network. After removal of OTUs whose relative abundances were lower than 0.01%, the correlation network of each treatment was calculated by SparCC (sparse correlations for compositional data) in Python v3.8. The parameter is set at five iterations, and the calculation of the two-tailed *P* value correction is based on 100 repeated samplings (92). The connections with a Spearman’s correlation coefficient (*r*) of >0.8 and significance at a *P* value of <0.05 were selected to construct co-occurrence networks in R3.5.0 software. Clustering and composition in the constructed networks were compared and plotted in Gephi-0.9.2.

The differences of pathogen abundance, probiotic traits, relative abundance of OTUs, physiological enzymatic activity, and numbers of NRPS and PKS genes were determined by ANOVA or *t* tests in IBM SPSS 20.0. Correlations between pathogen abundance, relative abundance of OTUs, physiological enzymatic activity, and NRPS or PKS gene numbers were determined by Spearman correlation. Analysis of similarities (ANOSIM) was performed to test the significant differences of microbial community composition according to Pseudomonas inoculation. The top 20 classified OTUs were plotted in RStudio by running the pheatmap package.

### Data availability.

The data sets generated for this study can be found in the NCBI Sequence Read Archive (SRA) with the accession number PRJNA931974. The partial 16S rRNA sequences of key OTUs and whole 16S rRNA gene sequences of isolated strains are given in the supplemental material. The three key Pseudomonas isolates (P8, S25, and S36) were deposited in the Gansu Culture Collection Center (http://jzk.gsmsc.cn/) in China under accession numbers GSICC31644, GSICC31643, and GSICC31642, respectively.
